# Trends in mortality for cancers, comparing multiple- and underlying-cause rates, in an English population 1979–1999

**DOI:** 10.1038/sj.bjc.6601633

**Published:** 2004-03-02

**Authors:** M J Goldacre, M E Duncan, P Cook-Mozaffari, M Griffith

**Affiliations:** 1 Unit of Health-Care Epidemiology, Department of Public Health, University of Oxford, Old Road, Oxford OX3 7LF, UK

## Abstract

In compiling official mortality statistics, rules for selecting the underlying cause of death have changed twice in the last 20 years in England. Mortality statistics for most types of cancer were not greatly affected, but there were significant effects on coding for cancers of colon, liver, breast, prostate, testis and bladder, and for lymphoma and leukaemia.

Until recently, only one underlying cause of death on death certificates has been routinely coded and analysed in most countries including England. By omitting causes of death that are not coded as the underlying cause, official statistics underestimate the extent to which specific diseases, such as specific cancers, contribute to the mortality of a population.

Interpretation of mortality statistics based only on underlying cause is also complicated by changes over time in the rules for selecting the underlying cause. Rules changed in England in 1984 and 1993 with different approaches to applying rule 3 of the International Classification of Diseases (ICD) (WHO, 1977; OPCS, 1985; Harvey *et al*, 1991; ONS, 1996). This rule specifies a number of diseases, including pneumonia, thromboembolism and heart failure, that should not be coded as the underlying cause of death, even if certified as such by the certifying doctor, if other diseases such as cancer are also on the death certificate; instead, rule 3 prescribes that the other disease should be selected as the underlying cause (WHO, 1977). The rule was rigorously implemented by the Office of National Statistics in England and Wales in the period 1984–1993 and then partially reversed (OPCS, 1985; ONS, 1996). In 1993, multiple-cause coding of death certificates was introduced as standard practice in England, but information about multiple-cause mortality for longer-term trends is not available nationally. We have used a regional database that included all certified causes of death from 1979 to 1999, not just those selected as the underlying cause, with the aim of comparing underlying- and multiple-cause-coded rates for cancers.

## METHODS

The former Oxford National Health Service Region covered a total population of about 2.5 million people. All causes of death on each death certificate in this population were coded for the years 1979–1999, for the Oxford record linkage study (Goldacre *et al*, 2000), using the 9th ICD revision codes. In each year, the underlying cause of death was selected and coded according to the prevailing national rules. Following convention, all certified causes of death – underlying cause plus causes elsewhere on the certificate – are termed ‘mentions’.

The database was searched for records with mentions of malignant neoplasms (ICD codes 140–208). We analysed the data in three time periods defined by changes to coding rules, 1979–1983, 1984–1992 and 1993–1999, to calculate the percentage of mentions that were coded as the underlying cause of death in each period. We tested changes in these percentages between time periods by *χ*^2^ tests on the numbers on which the percentages were based. We then calculated changes over time in annual mortality rates, standardised by applying the age-specific rates in the Oxford region population in each year to the European standard population. We calculated the average annual percentage change over time in mortality rates for mentions by fitting linear regression models to the logarithms of the death rates. We calculated changes in rates for mentions using every individual year. We calculated rates for underlying cause using individual years before and after the rule 3 changes, that is, omitting data for 1984–1992. We omitted these years to determine whether, if the discontinuity in the application of the rule was ignored, trends in mentions and underlying cause were similar.

## RESULTS

Table 1

**Table 1:** Mention-based mortality rates per 100 000 population; ratio of death with a mention of each cancer to death certificates with the cancer as the underlying cause of death; and average annual change in mortality rates with 95% confidence interval (95% CI in italics)

		**Ratio of mentions to underlying cause**								
**Cancer site or type (ICD codes; *N*=number of deaths with mentions)**	**Mention-based mortality rates per 100 000 1979–1999**	**1979–1983**	**1984–1992**	**1993–1999**	**Average annual % change in mortality rates and 95% CI: mentions, 1979–1999**			**Average annual % change in mortality rates and 95% CI: underlying cause 1979–1999 excluding 1984–1992**		
All cancer (140–208; *N*=128 068)	228.7	1.11	1.07***	1.10***	−0.8	−*0.9*	−*0.6*	−0.7	−*0.9*	−*0.6*

Tongue (141; *N*=365)	0.7	1.24	1.17	1.20	−0.1	−*2.1*	*1.9*	0.1	−*1.9*	*2.2*
Salivary glands (142; *N*=176)	0.3	1.07	1.08	1.19	−0.2	−*2.9*	*2.5*	−1.6	−*4.2*	*1.2*
Nasopharynx (147; *N*=127)	0.2	1.07	1.10	1.19	−3.3	−*5.7*	−*0.8*	−4.0	−*7.3*	−*0.5*
Oesophagus (150; *N*=4499)	7.9	1.09	1.05***	1.06	1.6	*0.9*	*2.2*	1.7	*0.9*	*2.6*
Stomach (151; *N*=6858)	12.4	1.08	1.06***	1.07*	−4.2	−*4.6*	−*3.7*	−4.1	−*4.5*	−*3.7*
Rectum (154; *N*=5318)	9.5	1.14	1.12	1.14*	−2.3	−*2.8*	−*1.7*	−2.4	−*2.9*	−*1.9*
Colon and rectum (153−154; *N*=15 572)	27.0	1.15	1.10***	1.13***	−1.4	−*1.8*	−*1.1*	−1.4	−*1.7*	−*1.0*
Liver and intrahepatic bile ducts (155; *N*=1256)	2.2	1.23	1.05***	1.07	2.5	*2.0*	*3.0*	3.5	*2.8*	*4.2*
Gallbladder and extrahepatic bile duct (156; *N*=729)	1.2	1.09	1.05	1.10	−4.5	−*5.7*	−*3.3*	−4.6	−*5.9*	−*3.3*
Pancreas (157; *N*=5375)	9.3	1.07	1.03***	1.04	−0.5	−*0.9*	−*0.2*	−0.4	−*0.8*	*0.1*
Digestive organs and peritoneum (159; *N*=997)	1.5	1.09	1.06	1.13**	8.1	*6.7*	*9.6*	7.9	*6.3*	*9.6*
Nasal cavities, middle ear, access sinuses (160; *N*=161)	0.3	1.19	1.05	1.09	−5.6	−*8.6*	−*2.4*	−4.8	−*8.6*	−*0.8*
Larynx (161; *N*=775)	1.5	1.22	1.28	1.44*	−0.6	−*2.0*	*0.9*	−1.7	−*3.5*	*0.2*
Trachea, bronchus and lung (162; *N*=28021)	53.3	1.06	1.05	1.06***	−2.3	−*2.6*	−*2.1*	−2.4	−*2.6*	−*2.1*
Pleura (163; *N*=373)	0.7	1.06	1.04	1.02	4.8	*3.0*	*6.6*	5.0	*3.4*	*6.7*
Bone and articular cartilage (170; *N*=282)	0.5	1.16	1.08	1.05	−1.2	−*3.8*	*1.5*	0.0	−*2.5*	*2.6*
Connective and other soft tissue (171; *N*=563)	1.0	1.12	1.06	1.09	1.7	*0.2*	*3.2*	1.9	*0.3*	*3.5*
Malignant melanoma of skin (172; *N*=1268)	2.3	1.09	1.07	1.06	2.3	*1.2*	*3.4*	2.7	*1.1*	*4.3*
Other cancer of skin (173; *N*=463)	0.8	1.62	1.39	1.56	−0.6	−*2.3*	*1.1*	0.0	−*1.7*	*1.8*
Female breast (174; *N*=13807)	44.3	1.14	1.10***	1.15***	−0.8	−*1.2*	−*0.5*	−0.9	−*1.3*	−*0.4*
Uterus, part unspecified (179; *N*=650)	1.9	1.30	1.13**	1.22*	−1.3	−*3.5*	*1.0*	−0.7	−*3.2*	*1.8*
Cervix (180; *N*=1367)	4.7	1.11	1.09	1.15*	−4.1	−*5.2*	−*3.0*	−4.2	−*5.5*	−*3.0*
Body of uterus (182; *N*=789)	2.4	1.15	1.16	1.20	−2.7	−*3.9*	−*1.4*	−3.2	−*4.5*	−*1.9*
Ovary and other uterine adnexa (183; *N*=3503)	12.0	1.05	1.03	1.06***	0.3	−*0.5*	*1.0*	0.2	−*0.5*	*0.9*
Prostate (185; *N*=8732)	35.0	1.39	1.19***	1.30***	1.5	*1.2*	*1.9*	1.9	*1.2*	*2.6*
Testis (186; *N*=134)	0.5	1.03	1.15	1.72**	−3.6	−*7.0*	*0.0*	−6.5	−*9.2*	−*3.7*
Penis (187; *N*=125)	0.5	1.39	1.06**	1.28*	−3.9	−*6.6*	−*1.0*	−3.4	−*6.2*	−*0.7*
Bladder (188; *N*=4759)	8.7	1.30	1.21**	1.26**	1.0	−*1.4*	−*0.6*	−0.9	−*1.3*	−*0.5*
Kidney (189; *N*=2338)	4.3	1.18	1.11**	1.15	1.8	*0.9*	*2.6*	1.8	*1.0*	*2.5*
Brain (191; *N*=2541)	4.9	1.04	1.02*	1.04*	0.5	−*0.2*	*1.2*	0.3	−*0.3*	*0.9*
Thyroid gland (193; *N*=299)	0.5	1.13	1.10	1.15	−1.3	−*3.5*	*1.0*	−2.1	−*3.7*	−*0.5*
Lymphosarcoma, reticulosarcoma (200; *N*=282)	0.5	1.12	1.20	1.19	−10.0	−*12.0*	−*8.0*	−10.1	−*12.5*	−*7.5*
Non-Hodgkin's lymphoma (200+202; *N*=3642)	6.4	1.19	1.12**	1.18***	1.7	*1.1*	*2.2*	1.6	*1.1*	*2.1*
Hodkin's disease (201; *N*=489)	0.9	1.20	1.20	1.40*	−4.2	−*6.0*	−*2.4*	−5.1	−*6.9*	−*3.3*
Other cancer of lymphoid and histiocystic tissue (202; *N*=3364)	5.9	1.21	1.11***	1.18***	2.9	*2.3*	*3.5*	3.0	*2.2*	*3.7*
Multiple myeloma and immunoproliferative cancers (203; *N*=2292)	4.0	1.20	1.12**	1.20***	0.4	−*0.3*	*1.0*	0.2	−*0.7*	*1.2*
Lymphoid leukaemia (204; *N*=1447)	2.5	1.39	1.39	1.56*	−0.6	−*1.9*	*0.8*	−1.5	−*3.4*	*0.4*

All cancers with more than 400 cases, or with significant changes in ratios or annual rates (cancers coded as those at ill-defined or unspecified sites excluded)

* *P*<0.05;

** *P*<0.01;

*** *P*<0.001.

shows the extent to which underlying cause statistics underestimate the total mortality ascribed to cancers. For example, in 1993–1999 mortality from all cancer was 10% higher, and that from breast cancer was 21% higher, than that judged from data on underlying cause alone.

**Figure 1:**
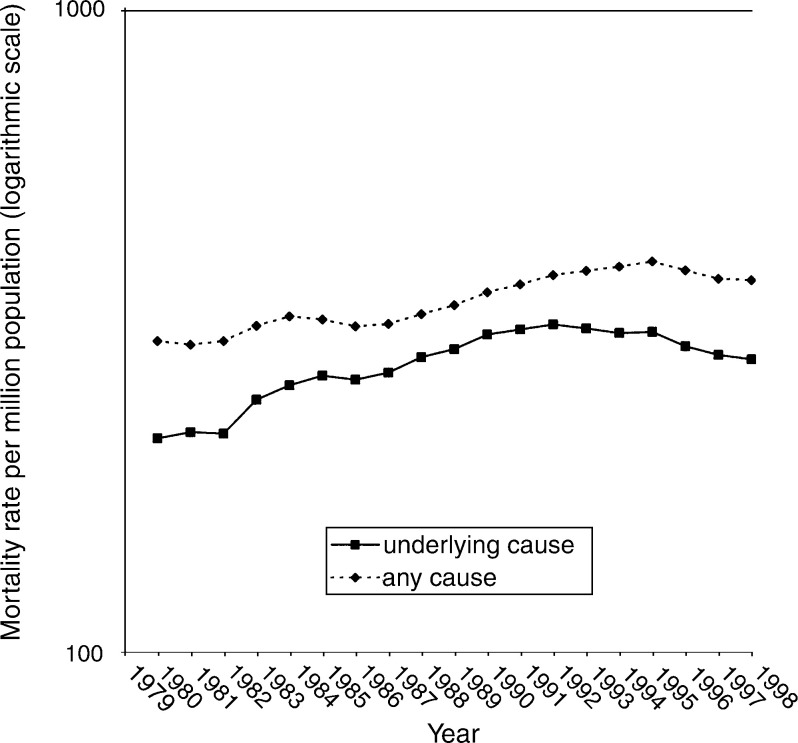
Mortality rates for cancer of prostate, expressed as age-standardised rates per million population and as 3-year moving averages around each calendar year, comparing prostate cancer coded as the underlying cause of death, and coded as a cause on any part of the death certificate.

Comparing periods with different coding rules (Table 1), the ratio of mentions to underlying cause showed statistically significant and noteworthy differences for cancer of the colon, liver, breast, prostate, testis, bladder, Hodgkin's disease, non-Hodgkin's lymphoma, lymphoid and myeloid leukaemia (we defined noteworthy as a significant change in the ratio of more than 5%). Statistically significant, but numerically smaller, changes in the ratios between the periods with different coding rules were seen for cancers of the oesophagus, pancreas and lung. Changes in the ratios coinciding with rule changes can be interpreted using prostate cancer as an example: between 1979–1983 and 1984–1992, the ratio of mentions to underlying cause decreased from 1.39 to 1.19, or, in other words, in the later of the two periods a higher percentage of certificates that included prostate cancer were coded with prostate cancer as the underlying cause. This meant that underlying-cause mortality rates for prostate cancer (the only rates available from routine statistics) exaggerated the rise from 1979–1983 to 1984–1992. The subsequent increase in the ratio of mentions to underlying cause, from 1.19 in 1984–1992 to 1.30 in 1993–1999, means that a lower percentage of cases with prostate cancer were coded with the disease as the underlying cause in the later than in the earlier of these two periods. In this example, the trends for underlying-cause and mentions converge comparing the first and second periods, and diverge comparing the second and third periods (Figure 1

).

Age-standardised death rates, expressed as mentions per 100 000 resident population, showed a significant increase over time for cancers of the oesophagus (in men only), liver, malignant melanoma, prostate, kidney, and non-Hodgkin's lymphoma. Significant decreases over time were found for cancers of the lip, stomach, colon, rectum, gall bladder, pancreas, lung (in men only), breast, bladder, Hodgkin's disease and non-Hodgkin's lymphoma. Omitting the middle time period, these trends based on underlying cause were similar to those based on mentions.

## DISCUSSION

The ratios of mentions to underlying cause show the extent to which statistics for underlying cause, alone, have underestimated the population toll of cancer mortality. For certain cancers, studies of trends in rates that use underlying cause only, starting or finishing in years between 1984 and 1992, may be misleading. In general, however, the impact of the rate changes on cancer was small. Particularly, if data for the years 1984–1992 are ignored, rates show similar trends whether analysed for mentions or for underlying cause. As we have shown, even over a period as short as two decades, many cancers show significant upward or downward trends.

Consideration needs to be given to the distinction between dying with a cancer and dying from a cancer. Particularly for cancers in which therapy has been successful, the certifying doctor may consider that the cancer, though appropriate to certify as a contributing cause, was not the underlying cause of death. As treatment improves, for some cancers it can be expected that there will be a trend away from certifying the cancer as the underlying cause of death in affected people who die. Testicular cancer and Hodgkin's lymphoma are examples (Table 1). Trends in mortality statistics for cancer, based on underlying cause alone, may be particularly difficult to interpret when the period studied coincides with changes in practice in the selection of the underlying cause.

## CONCLUSION

Multiple-cause coding of mortality in England has become routine since 1993. In analysing cancer mortality rates, consideration should be given to rates based on mentions as well as underlying cause, particularly when the analysis crosses periods of change to selection rules.
